# Case of bilateral hydronephrosis and spontaneous ureteral rupture as a rare complication of prostate biopsy

**DOI:** 10.1016/j.eucr.2022.102056

**Published:** 2022-03-17

**Authors:** Michal Korček, Lenka Barreto

**Affiliations:** Department of Urology, University Hospital Nitra, Špitálska 6, 949 01, Nitra, Slovakia

**Keywords:** Hydronephrosis, Ureteric rupture, Prostate biopsy, Urinary retention

## Abstract

Development of bilateral hydronephrosis and ureteric rupture has never been reported in the published literature so far. We describe a case of 51years old gentleman who developed this complication after*trans*-rectal ultrasound guided prostate biopsy. The patient was treated with bilateral double-J stent insertion, intravenous antibiotic therapy and recovered completely. There have been reports of hydronephrosis with or without ureteric rupture in the world literature. The causes reported have been such as malignancy, stones, retroperitoneal fibrosis, iatrogenic manipulation, trauma, degenerative kidney conditions and spontaneous causes. This could lead to development of retroperitoneal urinoma, urosepsis, abscess formation, infection and renal impairment.

## 1 Trauma and reconstruction

This research did not receive any specific grant from funding agencies in the public, commercial, or not-for-profit sectors.

## 2 Introduction

Our case of upper urinanary tract perforation is one of the rare complications of the prostate biopsy, which has never been reported before.

Minor complications such as pain or discomfort, haematuria or transrectal bleeding, infection, haematospermia and urinary retention are well known complications after prostate biopsy.

The complete lower urinary tract obstruction causing perforation of the upper tract due to swelling of the prostate after biopsy can happen and urologists should be aware of this.

## 3 Case report

A 51-years old fit and well man with elevated PSA (6,06 ng/ml) was admitted to our urological department for *trans*-rectal prostate biopsy. The prostate size was 30 g. We performed 12 core *trans*-rectal ultrasound guided biopsy. Patient had urethral catheter inserted post operatively. Six hours after operation the patient developed oliguria. The ultrasound KUB revealed normal kidneys and empty bladder. He remained stable and he was pain free. He was given 20mg Furosemide and 500mL Normal Saline intravenously and the urine output was 800mL per 24 hours.

First post-operative day the patient had rosē coloured urine with output of 1600 mL per 24 hours. Day two after operation the patient developed abdominal distension, left sided abdominal pain, decreased bowel sounds and anuria.

**Fig. 1:**
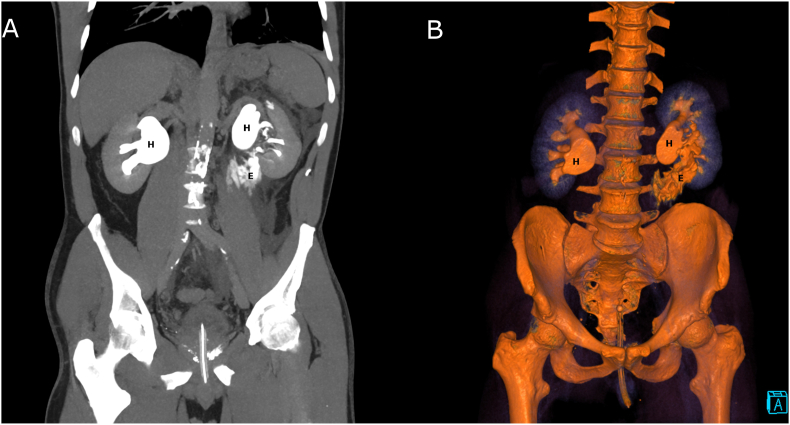
CT image; E, extravasation of the contrast from the ruptured ureter into the peri renal space; H, bilateral hydronephrosis. (A) CT coronal reconstruction. (B) CT 3D coronal reconstruction.

Contrast CT of the abdomen and pelvis was organised and revealed bilateral hydronephrosis, extravasation of the contrast from the left upper ureter and empty bladder (Fig. 1).

After the findings from the CT, the patient had cystoscopy with bilateral Double-J stent insertion. The cystoscopy didn't reveal any obvious pathology to explain the bilateral renal obstruction. He remained under intravenous antibiotic cover since the initial operation. He recovered well and was discharged three days after the stent insertion. The ureteric stents were removed one month after surgery. The histopathology of the prostate tissue showed benign prostatic hyperplasia.

## 4 Discussion

Prostate biopsy is an invasive procedure and carries the risks of complications. Raajimakers et al. reported in their large series of 5802 patients incidence of haematuria lasting longer than 3 days after 22.6% and haematospermia after 50.4% of the procedures.^1^ Complication such as infection can present as simple urinary tract infection, epididymitis or more severe complication in a form of sepsis. Other associated morbidities include rectal bleeding, transient lower urinary tract symptoms, erectile dysfunction, vasovagal episodes and urinary retention.^2^

Ureteric rupture is a rare condition and can be traumatic or non-traumatic in nature. The most common cause of traumatic ureteric rupture is iatrogenic trauma, followed by penetrating trauma, and occasionally blunt abdominal trauma. Nontraumatic ureteric rupture is caused by transmitted back pressure from a downstream obstruction which might be caused by ureteric stones, surgical ligature, or an abdominal or pelvic mass.^3^

The most common symptoms of ureteric rupture are sudden, severe, persistent lower abdominal pain with severe peritoneal irritation. In case of delay in initiating treatment, this may lead to serious consequences such as perinephric or retroperitoneal collection, abscess formation and urosepsis. Katz et al. described in their case report of iatrogenic ureteric rupture massive urinary leakage into the peritoneal cavity, resulting in abdominal compartment syndrome, respiratory distress, and anuria.^4^

Computerised Tomography (CT) urogram is considered the gold standard for optimal evaluation and diagnosis of ureteric rupture. In case of development of urinoma, it can provide accurate information about the location, the size of the urinoma, it's progression and information about any other complications.

In our case, we diagnosed ureteric rupture using the CT scan. In delayed-phase images, extravasation of contrast from the perforated left ureter was seen (Fig. 1).

Regarding management, many authors have resorted to open surgery for the treatment of spontaneous rupture and urinoma in the past. In the modern era, minimally invasive techniques such as endoscopic or percutaneous drainage can be used to manage complications. Stravodimos et al. reported successful treatment of ureteric rupture with the insertion of a Double-J ureteric stent. They achieved unobstructed urinary outflow, healing of the perforation, stabilization and gradual absorption of the urinoma.^5^ The incidence of late complications such as ureteric stricture, ureteropelvic stenosis, or peri-ureteric fibrosis in case of delayed treatment remains unknown.

In our case, there was no evidence of the factors that caused ureteric rupture other than the oedema of the prostate after the biopsy itself. Cystoscopy and CT did not show any other pathology. Acute bilateral distal ureteric outflow obstruction resulted in high intraureteric and intrarenal pressure. The ureteric wall failed to withstand the pressure and ruptured.

Prostate biopsy with subsequent swelling of the prostate may cause bilateral hydroureter and hydronephrosis and ureteral rupture in rare cases and the urologists should be aware of this possible complication.
